# Thermal constraints on the distribution of Japanese eel (*Anguilla japonica*) at its northern limit: Links to land use and geology

**DOI:** 10.1093/pnasnexus/pgaf384

**Published:** 2025-12-23

**Authors:** Kanta Muramatsu, Mari Kuroki, Yu-Lin K Chang, Kentaro Morita, Osamu Kishida

**Affiliations:** Graduate School of Environmental Sciences, Hokkaido University, Sapporo, Hokkaido 060-0810, Japan; Interfaculty Initiative in Information Studies, The University of Tokyo, Bunkyo, Tokyo 113-0033, Japan; Graduate School of Agricultural and Life Sciences, The University of Tokyo, Bunkyo, Tokyo 113-8657, Japan; Application Laboratory, Japan Agency for Marine-Earth Science and Technology, Yokohama, Kanagawa 236-0001, Japan; Atmosphere and Ocean Research Institute, The University of Tokyo, Kashiwa, Chiba 277-8564, Japan; Wakayama Experimental Forest, Field Science Center for Northern Biosphere, Hokkaido University, Hirai 559, Kozagawa, Higashimuro, Wakayama 649-4563, Japan

**Keywords:** climate change, distribution expansion, eel

## Abstract

Distributions of species can be shaped by large-scale geographic features present at broad spatial scales that control local environmental conditions. In species with complex life cycles, different local factors are expected to determine their distribution at different life stages in a sequential manner. Although the geographic distribution of fish with pelagic life stages (such as planktonic larvae transported by ocean currents) is likely determined by both pelagic stage and post-pelagic stage factors, the role of post-pelagic factors remains poorly understood. In this study, we examined the geographic and local factors that may influence the distribution of the Japanese eel (*Anguilla japonica*) at its northern range limit. We assessed the abundance of river-dwelling eels in 105 rivers across southern Hokkaido and analyzed statistical models that accounted for the potential recruitment of glass eels estimated through an oceanic transport simulation. Building on these models, we examined how local environmental conditions can influence eel abundance and identified the geographic features that likely regulate those local conditions. We found that Japanese eels were heterogeneously distributed at their northern limit. Our analyses suggest that Japanese eels were more abundant in rivers with watersheds characterized largely by farmland and urban areas and only minimally by volcanic geology, as these rivers tended to have warmer water temperatures during the feeding season. These findings suggest that global warming, in combination with urbanization, may facilitate the northward expansion of the Japanese eel, although in this region, the expansion may be constrained by the past volcanic activity.

Significance Statement This study explores how geographic and local factors may be related to the northern range limit of the Japanese eel (*Anguilla japonica*). The findings suggest that the distribution of fish with pelagic larval stages is shaped not only by oceanic transport during the pelagic phase but also by environmental conditions encountered after settlement. We found that Japanese eels were more abundant in rivers with higher summer water temperatures, with watersheds dominated by farmland and urban areas, compared to rivers with low summer water temperatures, with watersheds near volcanic regions. These findings highlight that Japanese eel stocks may be expanding northward due to global warming and urbanization, offering crucial insights for biodiversity conservation in the context of climate change.

## Introduction

The geographical distribution of a species is shaped by factors that operate at different spatial scales in a hierarchical manner (1, 2). The environmental conditions in areas where individuals live have a significant impact on individual fitness (3). The presence or absence of a particular species in a location is therefore largely determined by the spatial distribution of suitable and unsuitable environmental conditions (i.e. filtering factors). Thus, because the distribution of environmental conditions is shaped by geographical features present at broad spatial scales, the geographical distribution of a species is also shaped by those geographical features through their effect on local environments (4, 5).

Species with complex life cycles, defined as species that have discrete life stages during which the same organism differs in form or function (6), typically use multiple ecosystems across their life stages (7). The distribution of such species is governed by factors in each ecosystem that they inhabit during their dispersal and establishment phases (8). During the dispersal phase, individuals can be carried long distances by transport mediums such as wind, water currents, or highly mobile animals (9, 10). In contrast, during the establishment phase, the transported individuals settle in a specific environment, where they grow and mature (11, 12). Therefore, the dispersal range of a species is constrained by its dispersal potential during the dispersal phase (13). However, not all locations within this range are equally suitable: local environmental conditions encountered during dispersal can still vary considerably. Individuals may die or change their habitat if the local environmental conditions are detrimental (14, 15). Such environmental filtering during the establishment phase ultimately determines the realized distribution within the dispersal range (16). Hence, identifying the factors that limit the distribution of species at each life stage is crucial for understanding how the actual distribution of a species with a complex life cycle has been shaped.

Many marine fish with complex life cycles have pelagic stages (17). In early life stages, such as the egg and larval stages, such fish become widely dispersed across the ocean by currents because of their limited swimming ability (18). After this transportation phase, the pelagic eggs and larvae settle in local areas such as coral reefs and rivers, where they grow and mature; this settlement initiates the post-pelagic phase (18). While the range of dispersal is determined by the transport of individuals via water currents and by filtering processes acting on pelagic stage organisms (10, 19), the actual distribution within the dispersal range can be shaped by filtering processes acting at or after the time of settlement. However, previous studies that have investigated the local factors shaping fish distributions during the post-pelagic period have focused only on those factors determining their distribution at a small scale (e.g. food availability or structural complexity in coral reefs (20, 21)). To our knowledge, no studies have explicitly explored the factors that shape large-scale distributions (i.e. geographic distributions spanning tens to hundreds of kilometers).

**Fig. 1.**
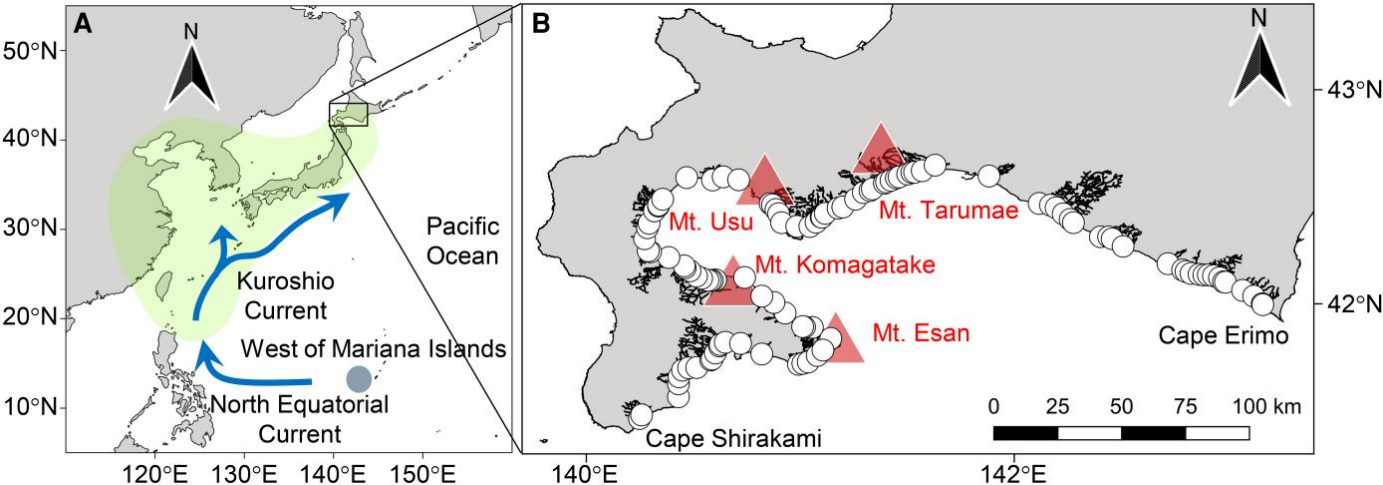
A) Map showing the location of southern Hokkaido, the spawning area of Japanese eel located west of the Mariana Islands (circle), and the ocean currents that transport the leptocephali (arrows). The distribution area of the Japanese eel after settlement (shading). B) Map of the study region showing the locations of the survey sites (open circles) and the target rivers. The westernmost point in the study region is Cape Shirakami, and the easternmost point is Cape Erimo. Shaded triangles indicate the locations of the four active volcanoes: Mt. Tarumae, Mt. Usu, Mt. Komagatake, and Mt. Esan.

The general question that arises here is whether geographic features shape the geographical distribution of fish with pelagic stages by affecting environmental filtering factors. To address this question, we explored factors shaping the distribution of Japanese eel (*Anguilla japonica*) at their northern limit. During pelagic stages (i.e. at the leptocephalus and oceanic glass eel stages), Japanese eels drift from west of the Mariana Islands in the western North Pacific Ocean, where they were spawned, to coastal areas of East Asia, including Taiwan, China, Korea, and Japan (22) (Fig. 1A). The newly hatched larvae are transported for several months, first by the North Equatorial Current and then by the Kuroshio Current (23). After metamorphosis of the leptocephali, the glass eels swim toward coastal areas and enter estuaries. They grow and undergo initial maturation in rivers over a period of several years to a decade or so during their post-pelagic stages (24). Environmental conditions vary among rivers, but rivers in close proximity tend to have more similar environments than rivers that are far apart, because neighboring rivers often share the same geographic features (25). Therefore, we hypothesized that geographic features shape the distribution of Japanese eels by determining habitat suitability through their effect on local environmental conditions.

This study focused on the Pacific side of southern Hokkaido, which is at the northern limit of recorded wild glass eel captures (26). Although there are a few records of yellow eels (growth phase eels) from this region, they are quite rare (27). These findings suggest that southern Hokkaido is the northern distributional limit of the Japanese eel. It is generally acknowledged that peripheral populations are more frequently exposed to harsh or extreme environments than core populations (28). Southern Hokkaido also exhibits a wide range of climatic characteristics (29) and land use patterns (30). Moreover, the region contains several active volcanoes, including Mt. Tarumae, Mt. Usu, Mt. Komagatake, and Mt. Esan (Fig. 1B), and geologically it showcases a diverse range of volcanic and sedimentary formations of various ages (31). Given the land use and geographic diversity, the region is well-suited for evaluating how environmental variation shapes eel distribution.

Through eel capture surveys conducted in over 100 rivers along the Pacific coast of southern Hokkaido (Fig. 1B), we identified the spatial distribution patterns of Japanese eels. We then used structural equation modeling (SEM), incorporating estimates of glass eel recruitment derived from a virtual larval-tracking simulation, to examine the potential influence of local environmental conditions on eel abundance. Finally, we applied a hierarchical SEM approach to explore how geographic features of rivers can shape eel abundance indirectly by affecting local environmental factors.

## Results

### Distribution pattern of Japanese eel

**Fig. 2.**
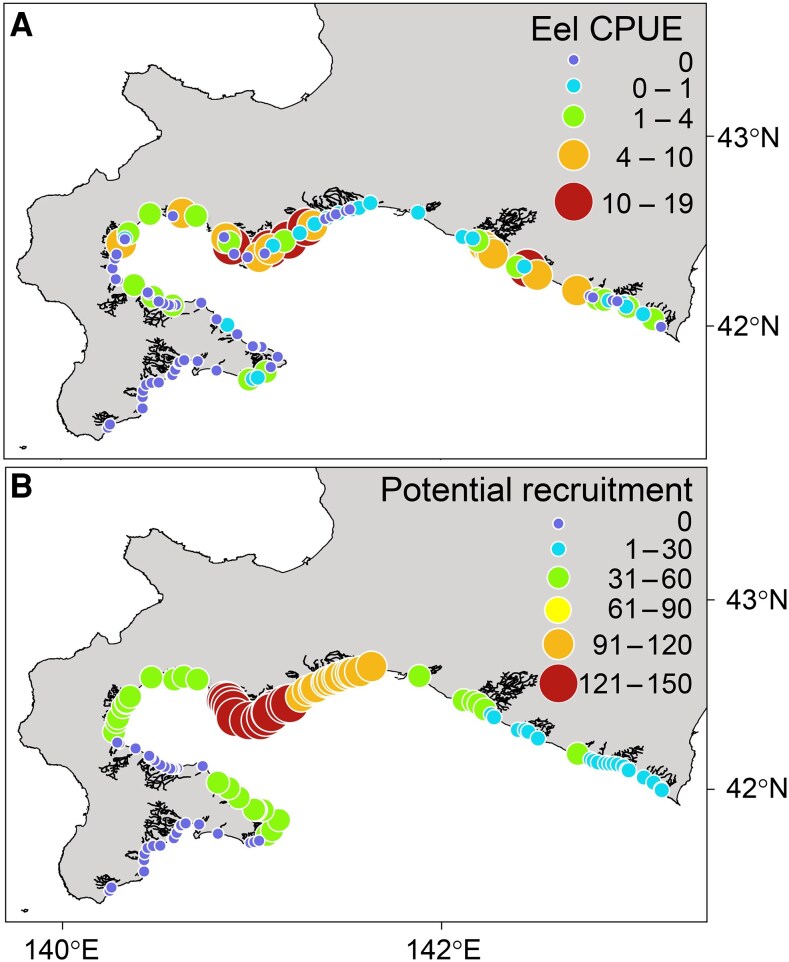
A) The spatial pattern of the eel CPUE across southern Hokkaido. The color and size of the circles represent the eel CPUE (individuals per hour). B) The spatial pattern of potential recruitment of glass eel recruitment across southern Hokkaido. The color and size of the circles represent the number of virtual eel larvae (v-larvae) estimated by the particle-tracking simulation to have entered the 0.5° longitude × 0.5° latitude area containing the mouth of each river.

To investigate the spatial distribution pattern of Japanese eel at their northern limit, we conducted capture surveys using electrofishing and estimated their relative abundance by calculating the catch per unit effort (CPUE). We captured a total of 222 Japanese eel individuals from 52 of the 105 rivers surveyed (average CPUE ± 1 SD, 2.1 ± 3.8 individuals perhour). The sizes of the captured eels varied widely (average total length ± 1 SD, 268.3 ± 100.6 mm; range, 79–701 mm). The eel CPUE exhibited a significant positive spatial autocorrelation (Moran's *I* = 0.17, *P* < 0.001), and this spatial distribution pattern appears robust, because it is highly consistent with the pattern estimated from capture surveys conducted in the previous autumn (32). This result reflects the fact that the multiple rivers in which we captured several eels and those in which we captured few or no eels tended to be spatially adjacent (Fig. 2A). The data from our capture survey strongly suggest that eels are distributed heterogeneously across southern Hokkaido, being present in some areas but absent in others.

### Factors affecting the eel abundance

**Fig. 3.**
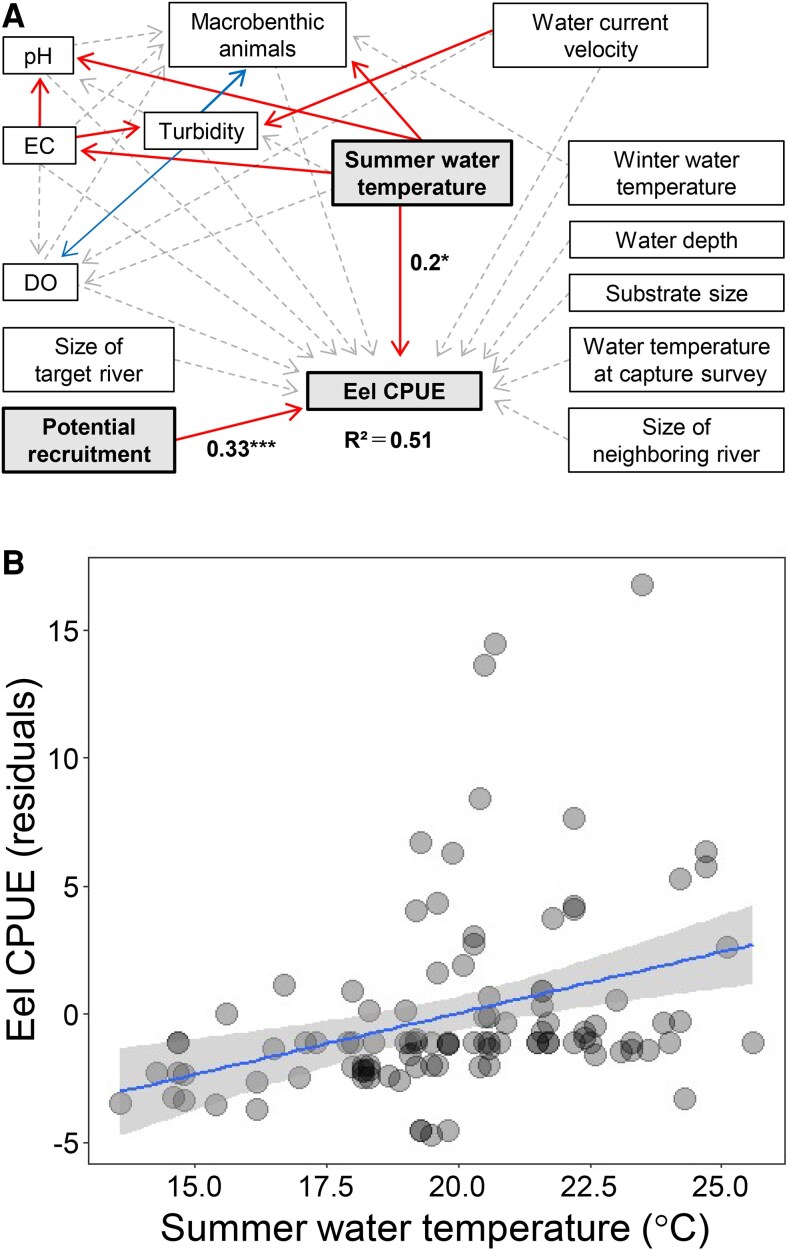
Potential effects of local factors on eel abundance (only summer water temperature was significant). A) Results of the structural equation modeling that incorporated eel CPUE, the 13 environmental variables, and the degree of glass eel recruitment. The red arrows represent positive paths, and the blue arrow represents a negative path. The dashed arrows represent nonsignificant paths (*P* ≥ 0.05), and the solid arrows represent significant paths (*P < 0.05, **P < 0.01, ***P < 0.001). Standardized partial regression coefficients are shown only for variables that significantly affected the eel CPUE. B) Relationship between summer water temperature and eel CPUE, represented by the residuals of eel CPUE after accounting for potential recruitment. The method for calculating these residuals is shown in Fig. S2. The solid line represents the linear regression (*y* = 0.05*x* + 19.27, *R*^2^ = 0.065, *P* = 0.0085). Because this reduced model omits indirect pathways, the *R*^2^ and *P* values differ from those in panel (A).

We assumed that the favorability of river conditions determines eel abundance in rivers through three key processes: (i) river ascendance of glass eels that have reached the coast (narrow-sense recruitment), (ii) The subsequent survival of river-resident eels (i.e. elver, yellow eel, and silver eel) that have migrated upstream, and (iii) movement of eels between-rivers and between river and coastal area. We hypothesized that abiotic and biotic environmental factors in rivers directly or indirectly may influence these potential processes and ultimately determine eel abundance (Fig. S1). To infer which direct and indirect pathways determine eel abundance, we employed piecewise SEM, with CPUE (i.e. the number of eels captured per hour during electrofishing surveys) as the response variable. As both direct and indirect explanatory variables, we included ten environmental factors of the focal river, such as river size, average summer and winter water temperatures, substrate size, and prey availability (Table S1). The model also incorporated the relative number of glass eels reaching the coast (i.e. potential recruitment of glass eels), estimated through a virtual glass eel tracking simulation, as a covariate (Fig. 2B). In addition, we included the size of neighboring rivers—which may influence both glass eel recruitment and subsequent inter-river movement during later life stages—as well as water temperature at the time of the capture survey, which could confound CPUE estimates by affecting capture efficiency (33). The resulting model fitted well to the data (Fisher's *C* = 46.25, *P* = 0.82) and explained 51% of the total variation in the eel CPUE (coefficients of determination *R*^2^ = 0.51). The SEM analysis that considered potential recruitment of glass eels (standardized partial regression coefficient *β* = 0.33, *P* < 0.001) as a covariate showed that among the environmental variables, only the average water temperature in summer (*β* = 0.27, *P* = 0.013) had a significant positive direct effect on eel CPUE (Fig. 3). We found no significant relationship between other explanatory variables including water temperature at the capture survey and eel CPUE (Table S2). These results suggest that the summer water temperature of the rivers was the only local variable with a detectable positive association with among-river variation in CPUE after accounting for potential recruitment.

**Fig. 4.**
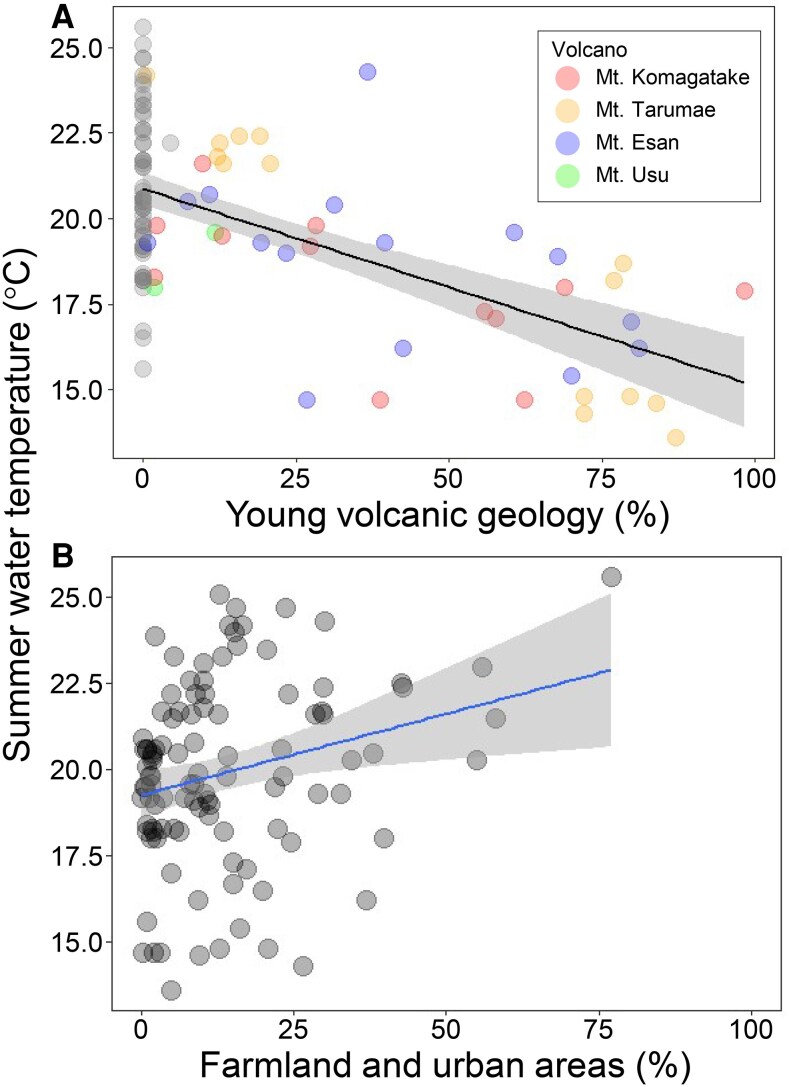
A) Relationship between the percentage of young volcanic geology and average summer water temperature (*y* = −0.06*x* + 20.87, *R*² = 0.35, *P* < 0.001). Colors indicate source volcanoes. B) Relationship between the combined percentage of farmland and urban areas and average summer water temperature (*y* = 0.05*x* + 19.27, *R*² = 0.07, *P* = 0.009). Shaded areas denote 95% confidence intervals.

To explore the geographic features potentially influencing summer water temperature, we analyzed the association of five geographic variables (including land use, geology, and climatic variables) in watersheds by multiple regression. The proportions of old (pre-Quaternary) volcanic geology (*β* = −0.29, *P* < 0.001) and young (Quaternary) volcanic geology (*β* = −0.66, *P* < 0.001) in the watershed were significantly negatively associated with the summer water temperature, whereas the proportion of farmland and urban area (*β* = 0.24, *P* = 0.003) in the watershed was significantly positively associated with it (Table S3; Fig. 4). We found nonsignificant relationships between precipitation and solar radiation and the summer water temperature (Table S3). These results suggest that watershed geology and land use shaped the variation in summer water temperature of the rivers in this study region.

**Fig. 5.**
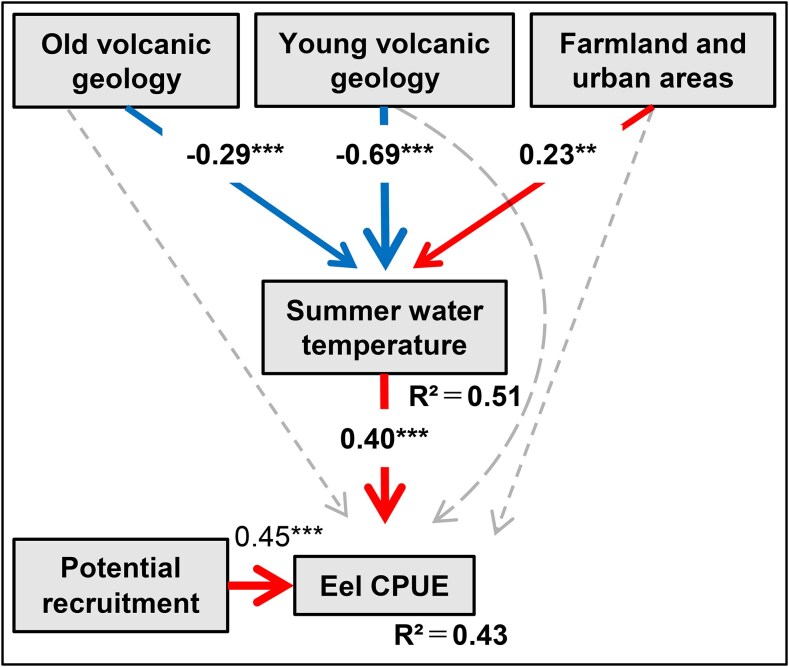
Results of the integrative structural equation modeling that incorporated eel CPUE, summer water temperature, the degree of glass eel recruitment, and geographic variables. The red arrows represent positive paths, and the blue arrows represent negative paths. Dashed lines represent nonsignificant paths (*P* ≥ 0.05), and solid lines represent significant paths (*P < 0.05, **P < 0.01, ***P < 0.001). Standardized partial regression coefficients are shown only for variables that significantly directly or indirectly affected the eel CPUE.

To examine how geographic features may influence eel abundance, we employed hierarchical SEM that incorporated eel CPUE as a response variable, three geographic variables as predictor variables, summer water temperature as a mediator variable, and the estimated potential recruitment of glass eels as a covariate (Fig. S3). The resulting hierarchical model fitted well to the data (Fisher's *C* = 0.35, *P* = 0.16) and explained 43% of the eel CPUE (*R*^2^ = 0.43) and 51% of the August water temperature (*R*^2^ = 0.51). The SEM analysis that considered the effect of glass eel recruitment (*β* = 0.45, *P* < 0.001) showed that proportions of old volcanic geology (*β* = −0.29 × 0.40 = −0.12) and young volcanic geology (*β* = −0.66 × 0.40 = −0.26) had significant negative indirect associations, whereas the proportion of farmland and urban area (*β* = 0.23 × 0.40 = 0.092) had a significant positive indirect association on eel CPUE through the summer water temperature (*β* = 0.40, *P* < 0.001) (Fig. 5). However, the direct paths from these geographic variables to eel CPUE were not significant; this result suggests that the effect of these geographic variables through the mediation of local environmental conditions other than summer water temperature was not significant (Table S4). Together, these results suggest that geology and land use may influence eel distribution within their dispersal range through their association with river water temperature.

## Discussion

Previous studies on the distribution of species with pelagic stages have shown that the pelagic stage factors limit the dispersal range of those species (e.g. (10, 19, 34)). However, the post-pelagic factors that limit the distribution of fish within their dispersal range are poorly understood. Here, we examined how the distribution of Japanese eels is associated with environmental conditions in the rivers after the pelagic stages, and how the geographic features may influence those environmental factors. First, we found that the abundance of Japanese eels in rivers varied greatly among local areas in the study region. Next, we showed that the spatial variation in the abundance of Japanese eels appeared to be related to the water temperature of rivers, which in turn was likely affected by geographic features such as human land use and natural geological characteristics.

Low-temperature stress has been considered a key factor limiting species distributions at the high-latitude edges of their ranges (35, 36). Our findings suggest that this may also apply to Japanese eels. Summer water temperature was the only local factor that accounted for inter-river variation in eel abundance. Specifically, rivers with higher summer water temperatures tended to support higher abundances of Japanese eels. Previous studies have shown that glass eels prefer warmer freshwater (18 °C) over colder freshwater (10 °C) when entering rivers (37), and that the foraging activity of Japanese eels drops sharply at temperatures below 15 °C (38). Therefore, the lower abundance of eels in rivers with lower summer water temperatures may have resulted from low-temperature stress, through a decrease in the entry of glass eels into rivers and/or suppressed foraging activity after entry, which could in turn lower survival or promote emigration to neighboring rivers or coastal areas.

We found no evidence that likely environmental factors other than summer water temperature affected the abundance of Japanese eels (Fig. 3A). One possible factor influencing the distribution of fish species may be winter water temperature, because extreme cold can disrupt the physiology of organisms (39). The nonsignificant relationship between winter water temperature and eel abundance may be explained by the negative correlation between summer and winter water temperatures in the survey region (Table S2). This pattern arises from contrasting seasonal water temperature trends across areas with different geological characteristics. The geology of this region is composed of two mutually exclusive components—volcanic and sedimentary formations—such that areas with a high proportion of volcanic geology have a correspondingly low proportion of sedimentary geology. As described below, compared to rivers in sedimentary regions, those in volcanic regions tend to have lower water temperatures in summer and higher temperatures in winter (40). In volcanic rivers, the potentially positive effects of relatively high winter water temperatures on eel survival and growth may be offset by the negative effects of low summer temperatures. In contrast, in sedimentary rivers, the negative effects of relatively cold winter temperatures may be mitigated—or even outweighed—by the positive influence of higher summer water temperatures, potentially contributing to higher eel abundance in those rivers. Although an alternative factor affecting eel abundance may be prey resources, in our analyses, the relationship between macrobenthic animals and eel abundance was not significant. This result may be due to the low abundance of Japanese eels in southern Hokkaido. In fact, Kasai et al. (41) reported that the environmental DNA (eDNA) of Japanese eel is barely detectable in rivers in southern Hokkaido. They detected eel eDNA in only one river and only at an extremely low concentration compared to eel eDNA in rivers on Honshu Island, which is the major distribution area of Japanese eel in Japan. Although past research has experimentally shown that extreme dissolved oxygen and pH conditions can have harmful effects on eel physiology and behavior (42, 43), we found no significant effects of related variables here, likely because extreme conditions are absent in the rivers in our survey region.

Our results suggest that the proportions of volcanic geology in a watershed had a negative effect on summer water temperature, which in turn, may have affected the abundance of eel (Fig. 5). Furthermore, the potential negative effect of young volcanic geology on water temperature was stronger than that of old volcanic geology. In fact, few or no eels were captured in areas dominated by young volcanic geology, such as around Mt. Tarumae, Mt. Esan, and Mt. Komagatake (Fig. S4). The average number of Japanese eels captured per hour was only 0.57 in the 10 rivers flowing around Mt. Tarumae, where the watersheds are characterized by young volcanic geology, even though this area was estimated to receive many of the glass eel arrivals. The permeability of the geology explains the relationship between water temperature and geology. The permeability coefficient of volcanic geology is higher than that of other geologies, such as sedimentary geology (44). Young volcanic geology, in particular, has an extremely high permeability coefficient (45). Precipitation infiltrates into highly permeable geologies rapidly; as a result, the spring-fed rivers in such areas receive relatively low-temperature water in summer and relatively high-temperature water in winter (40). Conversely, rivers flowing through areas with a less permeable geology (i.e. sedimentary geology) are fed primarily by surface runoff, which easily becomes heated in summer. The distribution and composition of geologies, which are determined by the locations of volcanoes and the age of the volcanic activity, may thus in turn determine the spatial distribution of river water temperature and, indirectly, the distribution of Japanese eels.

Our results also suggest that farmland and urban areas provide warmer environments for Japanese eel (Fig. S4), where a thermal refugium is defined as an area that buffers a species from unfavorable, sustained climatic thermal conditions (46). Some empirical studies have provided evidence showing that both urbanization and agricultural activity can elevate stream water temperatures. For example, Grey et al. (47) found that even small amounts of urban land cover can significantly increase stream temperatures through mechanisms such as increased surface runoff from impervious surfaces and the efficient transfer of heat via stormwater infrastructure. Agricultural areas can have similar effects, as runoff and return flows from irrigation may introduce heated water into streams (48). In general, organisms inhabiting high-latitudinal areas at the limits of their distributional range are exposed to high levels of low-temperature stress, which has been reported to limit the distribution of species in such regions (49, 50). Thus, human activities might create rivers that are warmer and therefore provide more suitable habitats for organisms living in extreme environments at the high-latitude edges of their distributional range (51), even though such altered environments can simultaneously be detrimental to cold-adapted species that originally inhabited these areas (52).

Our findings have significant implications for the conservation of the Japanese eel, a fish with a high commercial value that is also endangered. Global warming is causing the distribution of many species to shift toward higher latitudes, along with the shift in the range of temperatures suitable for each species (50). The fact that in this study water temperature was a prospective limiting factor in the distribution of Japanese eels suggests that this species too may be expanding its range to higher latitudes in response to global warming. Another key finding of this study is that water temperature can be determined by both human land use and geological features. In other words, human activities, particularly land use, under the constraint of geology resulting from past volcanic activity, may facilitate the northward expansion of Japanese eel under global warming.

We highlight that identifying the post-pelagic stage filtering factors of a fish is crucial for understanding how the actual distribution of a fish with pelagic stages is shaped and for predicting its spatial distribution pattern. Although we focused on the entire post-pelagic period, there may be several post-pelagic stages, such as the settlement stage and subsequent growth stage (18). Similarly, the pelagic period can comprise several stages. Different factors acting on eels at different pelagic or post-pelagic stages would determine the survival and habitat selection of individuals. To gain a comprehensive picture of the mechanisms that shape the distribution of fish with pelagic stages, the factors affecting fitness components, such as survival and growth, at each pelagic and post-pelagic stage, need to be investigated at high resolution.

## Materials and methods

### Field survey region

Our eel capture survey and assessment of river environmental conditions targeted 105 relatively small rivers flowing into the Pacific Ocean from southern Hokkaido (between Cape Shirakami and Cape Erimo) (Fig. 1B). Because of the challenges involved in conducting quantitative capture surveys in large rivers, which may be wide and deep, we excluded any rivers with a maximum width in the lowermost reaches exceeding 50 m. Previous studies conducted in the primary distribution area of Japanese eels, such as on Honshu, Kyushu, and Shikoku islands, have shown that eel density increases with proximity to a river's estuary (53, 54). Therefore, we conducted our survey in only the lowermost reach of each river (mean ± SD distance from the river mouth to the lower end of the sampling site, 325.8 ± 490.5 m, *n* = 105).

### Eel capture survey

To examine the distribution pattern of Japanese eels in southern Hokkaido, we conducted field surveys in 105 rivers between June and August 2023. Eels were captured using a backpack electrofishing unit (100 to 400 V DC; Model 12B, Smith-Root, Inc., Vancouver, WA, USA) in combination with 3-mm-mesh dip nets (30 cm wide). Surveys were conducted in the lowermost reaches of the rivers, specifically targeting habitats preferred by eels—areas with sufficient depth (>15 cm), slow flow velocity (<25 cm s⁻¹), rocky substrates, and abundant vegetation. Because our aim was to efficiently assess relative eel abundance rather than estimate precise densities, we employed a single-pass electrofishing method rather than a multiple-pass approach, allowing for broader spatial coverage. Each river was surveyed for a minimum of 1 h. The anode pole of the backpack electroshocker was moved at a consistent speed of 15 cm s^−1^. Given that the effective electric field extended ∼50 cm in radius from the tip of the anode pole, this resulted in a survey coverage of ∼540 m^2^ h^−1^. Each captured eel was photographed, and total length was later measured from the images using the software ImageJ (55). After photographing, all the eels were released. To avoid recapturing released individuals in the same survey (i.e. double-counting), all eels were released immediately after photographing at points downstream of the sampling reach, and we visually confirmed their refuge locations outside the reach.

As an index of eel abundance, we calculated CPUE by dividing the number of eels captured by the total survey time in hours (Table S1). Before each survey, we measured water temperature at the site, as it may influence the capture probability of eels (33).

### Simulation to estimate the potential recruitment of glass eels

We estimated the relative abundance of glass eels reaching the vicinity of each river mouth as the potential recruitment in each river by conducting a simulation of virtual eel larvae (v-larvae) transport based on an ocean circulation model. We used the ocean currents from a data-assimilative ocean circulation model, the Japan Coastal Ocean Predictability Experiment 2 (JCOPE2M) (56), which has a horizontal resolution of 1/12° (8 to 9 km) and 46 vertical layers. JCOPE2M has previously been used to study the impact of ocean circulation on marine ecosystems (57, 58), including the study of the transport of glass eels in our study region (32). We applied a 3D particle-tracking scheme based on the fourth-order Runge–Kutta method (59) and the ocean currents provided by JCOPE2M to simulate the movement of virtual eel larvae (v-larvae).

In a recent survey, eels aged between 1 and 10 years or older, as estimated by using otoliths, were discovered in southern Hokkaido (27). We therefore used 10 years of ocean data, from 2014 to 2023, to simulate the movement of the v-larvae. The region and time of the release of v-larvae were chosen on the basis of the historical eel larvae catch off northernmost northeast Japan (60). Approximately 4,000 v-larvae were released in the Kuroshio–Oyashio Confluence Zone (142.3° to 144.8°E, 39° to 41°N) each year. According to Tsukamoto (61), glass eels are found in rivers close to the release point in April, and they are found in the Abira River in our survey region of southern Hokkaido from May to July (32). Therefore, we released v-larvae on April 1 of each year from 2014 to 2023 and tracked them until July 30 of each year. Following Chang et al. (58), the v-larvae were programmed to swim horizontally at a speed of 0.15 m s^−1^. Diel vertical migration was also considered by the particle-tracking scheme and was set to 20 m during the night and to 300 m in the daytime.

### Survey of environmental conditions in the rivers

Past studies have shown that water temperature (37, 38), chemical characteristics (42, 43), and hydrodynamic characteristics (62), as well as prey availability (63), influence the survival and habitat selection of eels in rivers. Considering these previous findings, we measured the following 11 environmental variables in each river: summer and winter water temperature, pH, dissolved oxygen concentration (DO), electrical conductivity (EC), turbidity, density of macrobenthic animals as potential prey items, water depth, substrate size, water current velocity, and size of the watershed area.

We monitored the water temperature in each river by installing temperature data loggers (Elitech RC-5) that we had made waterproof. The data loggers, which were installed within or in close proximity to the capture survey site in each river, monitored the water temperature for 8 months, from January to August 2023. The lowest water temperature was observed in January, and the highest was observed in August. Therefore, in this study, we used the median temperature in January as the winter temperature and the median temperature in August as the summer temperature (Table S1). The recovery rate of the data loggers was 90% (95 of 105) in summer and 64% (67 of 105) in winter. If we were unable to retrieve a data logger from a river, we substituted water temperatures measured in a neighboring river with similar geographic and climatic characteristics for the missing data. This interpolation method is appropriate, because the reliability of this method was confirmed in an additional analysis focusing on rivers where logger retrieval was successful (95 rivers in summer and 67 rivers in winter). In the additional analysis, assuming missing data for each river, we used the above method to substitute the water temperatures, and a strong correlation was observed between the substituted values and actual measurements (the Spearman's correlation coefficients: 0.73 in summer and 0.61 in winter).

We measured the water depth, substrate grain size, and water current velocity as indicators of hydrodynamic characteristics. We selected a 50 m section parallel to the riverbanks within the capture survey site and drew 10 longitudinal transects perpendicular to the banks at even intervals. Then we measured the hydrodynamic characteristics of the river at both ends (i.e. the deepest water depth within 10% of the distance from the bank of the transect length) and in the middle of each transect. The water current velocity was measured at 60% water depth with a propeller-type current meter (VR-201, Kenek Co., Tokyo, Japan) by recording the average velocity during 5 s. We visually determined the representative substrate grain size and classified it according to Kumai et al. (62) as follows: 1 = sand or silt; 2 = gravel (major axis = 2 to 16 mm); 3 = pebble (17 to 64 mm); 4 = cobble (65 to 256 mm); 5 = boulder (>256 mm); and 6 = concrete or bedrock. We used the average water depth, water current velocity, and substrate size at 30 points in a river as the environmental variables for that river (Table S1).

After measuring the hydrodynamic characteristics, we surveyed the potential prey community. Within the capture survey site, three 5 m × 2 m sections were selected that included pools, and we captured all fish and large crustaceans (shrimps and crabs) using a backpack electrofishing unit and dipnets. After anesthetizing the collected animals with phenoxyethanol (ca. 0.5 mL L^−1^ water), we spread them out on a white tray (30 cm × 20 cm × 4.5 cm) and took photographs. Later, we counted macrobenthic animals, which included Gobiinae, Cobitididae, Caridea, Brachyura, Cottidae, Petromyzontidae, Gasterosteidae, and Pleuronectidae. We used the sum of the macrobenthic animals collected in the three sections as an index of the density of macrobenthic animals (Table S1).

We measured the chemical characteristics of the river, including pH, DO, EC, and turbidity, with a portable multi-parameter water quality meter (WQC-24, DKK-TOA Co., Tokyo, Japan) within or in close proximity to the capture survey site during early July, when the water levels were low (Table S1).

As an indicator of river size, we used watershed area. We first used QGIS v. 3.32.3 (https://qgis.org/en/site/) to calculate the watershed area of each target river, based on the 10-m Digital Elevation Model (DEM) published by the Geospatial Information Authority of Japan. All maps in this article were created using the Basic Geospatial Information provided by the Geospatial Information Authority of Japan (GSI).

### Survey of the geographic features of the rivers

To identify the geographic features that influence the spatial heterogeneity of summer water temperature—which was the only factor that significantly explained variation in eel abundance—, we focused on geology, land use, air temperature, precipitation, and solar radiation, as likely to be responsible for water temperature (64).

Sedimentary and volcanic geologies were dominant in the study region (31). Volcanic geology, which was found in close proximity to active volcanoes, was further classified into young (Quaternary age) and old (pre-Quaternary) volcanic geology. Areas with volcanic geology are known for their higher precipitation infiltration capacity (i.e. higher permeability) compared with areas with sedimentary geology (44). As a result, in areas dominated by volcanic geology, especially young volcanic geology, the water temperature in spring-fed rivers is lower in summer and higher in winter, compared with areas where sedimentary geology is predominant (40). Therefore, we used the areal proportion of sedimentary, old volcanic, and young volcanic geology in each watershed as an index of the watershed geology that potentially determined the water temperature in the river. This calculation was based on the 1:200,000 Land Classification Basic Survey (surface geology) map for Hokkaido from the Japan Ministry of Land, Infrastructure, Transport and Tourism (MLIT) (65) (Table S1).

Rivers flowing through farmland and urban areas tend to warm up more easily than those flowing through forests (48). Using 100-m mesh land use classification data for Hokkaido from MLIT (66), we calculated the areal proportions of farmland + urban area and forest in each watershed (Table S1). We used 1-km-mesh climate data from MLIT (67), which had been estimated and calculated based on the average values of meteorological data observed by Japan Meteorological Agency automated meteorological data acquisition system stations over the 30 years prior to 2020. The climatic variables of each river were determined as the average air temperature, precipitation, and global solar radiation in August in each river mesh that included the survey site.

We observed high correlations between the areal proportion of sedimentary geology and both areal proportions of old (Spearman's correlation coefficient = −0.67) and young volcanic geology (Spearman's correlation coefficient = −0.52), between the areal proportions of farmland + urban area and forest (Spearman's correlation coefficient = −0.97), and between air temperature and solar radiation (Spearman's correlation coefficient = 0.88). Therefore, to avoid multicollinearity in subsequent analyses, we excluded sedimentary geology, forest, and air temperature as geographic variables. All geospatial analyses were performed with QGIS 3.32.3 software (https://qgis.org/en/site/).

### Ethics

All animal surveys were performed in compliance with the Japanese regulations on animal welfare and were approved by the relevant institutional ethics committee (Approval No. 5-2, the Animal Experiment Committee of FSC of Hokkaido University).

### Statistical analyses

To investigate whether the spatial pattern of eel abundance in southern Hokkaido was random, we performed Moran's *I* test using the coordinates of the starting points of eel capture surveys and the eel CPUE. Using Moran's *I* statistic, we assessed whether any positive or negative spatial autocorrelation occurred in eel abundance. A significant positive spatial autocorrelation indicates that eel abundance tends to show similar values across neighboring rivers, providing statistical evidence for a nonrandom spatial distribution.

We hypothesized that the environmental factors might interact with each other, and that each environmental variable directly or indirectly influenced eel abundance. To examine the environmental conditions that can directly or indirectly affect eel abundance, we employed piecewise SEM. Piecewise SEM was applied in an exploratory manner to evaluate how the observed covariance structure among variables is consistent with directionally specified relationships based on ecological plausibility, rather than with an a priori hypothesis (see (68)). We constructed a model that incorporated eel CPUE, the 12 environmental variables of the focal river, including water temperature at the capture survey, one variable of the neighboring river (i.e. watershed size of the nearest river), and the degree of potential glass eel recruitment. We assumed direct paths between these variables based on ecological knowledge (see Fig. S1 for further details). Winter water temperature, EC, turbidity, and river size were log-transformed (base e) to ensure normal distributions. All potentially causal relationships between variables in the SEM were analyzed using generalized linear models (GLMs). For GLMs with eel CPUE as the response variable, a negative binomial distribution was assumed, whereas Gaussian distributions were assumed for models with other response variables. The goodness of fit of the SEMs was evaluated by using Shipley's test of directional separation (69) to confirm an adequate model fit (*P* > 0.05). All regression coefficients for each pathway were standardized to assess the significant effects of the environmental variables on eel abundance. For reference, because our CPUE data contained many zero values, we tested a zero-inflated model in addition to the current model. However, due to multicollinearity, the zero-inflated model became statistically unstable and could not be applied.

Multiple regression was employed to reveal the geographic features controlling the local factors determining eel abundance. We used a model with five geographic variables as predictor variables and summer water temperature as the response variable, because the piecewise SEM result revealed summer water temperature to be the only variable that significantly (*P* < 0.05) affected eel CPUE (see Results). We thus assessed the significant geographic variables responsible for the summer water temperature.

We hypothesized that the three geographic features that had a significant (*P* < 0.05) effect on the summer water temperature (see Results) indirectly influenced eel abundance. To disentangle the complex causal relationships between geographic features and eel CPUE as mediated by summer water temperature, we constructed a hierarchical SEM that integratively incorporated eel CPUE as the response variable, the three geographic variables as predictor variables, summer water temperature as a mediator variable, and the degree of glass eel recruitment as a covariate into a single causal network (see Fig. S3). We then assessed the fit of the model and the significant effects of geographic features on eel abundance. All statistical analyses were conducted in R version 4.2.2 using the packages “ape,” “base,” “dplyr,” “lavaan,” “MASS,” “Matrix,” “performance,” “piecewiseSEM,” “psych,” and “stats” (70). The dataset and R scripts supporting the findings of this study are available on Figshare at https://figshare.com/s/47455a4bae6d7f14c2c6.

## Supplementary Material

pgaf384_Supplementary_Data

## Data Availability

The dataset and R scripts supporting the findings of this study are available on Figshare at https://figshare.com/s/47455a4bae6d7f14c2c6.
