# Supplementary material for: Thermal constraints on the distribution of Japanese eel (Anguilla japonica) at its northern limit: Links to land use and geology
Source: PNAS Nexus. 2025 Dec 23;4(12):pgaf384. doi: 10.1093/pnasnexus/pgaf384 (PMC12723659; doi:10.1093/pnasnexus/pgaf384)
Supplement: pgaf384_Supplementary_Data [file pgaf384_supplementary_data.pdf]

Supplementary Materials

Thermal constraints on the distribution of Japanese eel (*Anguilla japonica*) at its northern limit: links to land use and geology

Kanta Muramatsu[1], Mari Kuroki[2,3], Yu-Lin K. Chang[4], Kentaro Morita[5], Osamu Kishida[6*]

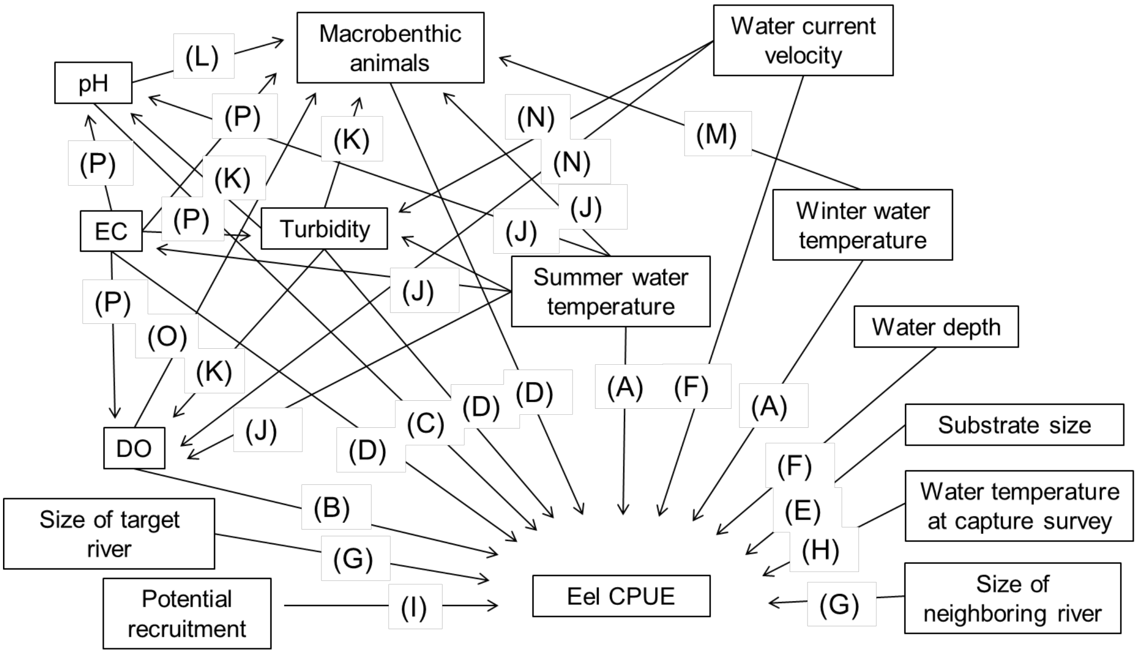

Fig. S1. Hypothesized causal network relationships among eel CPUE, 10 environmental variables, and the degree of glass eel recruitment. The brief explanations below of model paths (A) to (N) are based on ecological knowledge.

(A) Foraging activity of Japanese eels decreases when water temperature is low (15 °C) (Fukuda et al., 2009), and an extremely cold water temperature (5 °C) causes the death of glass eels (Han et al., 2012). Furthermore, glass eels selectively enter areas with warmer freshwater (Edeline et al., 2006). Thus, we expected eel abundance to be lower in rivers where seasonal water temperatures (in winter or summer) are lower.

(B) The foraging activity of eels decreases when dissolved oxygen (DO) is low (5 mg L^{-1}) (Rowchai et al., 1986). Thus, we expected eel abundance to be lower in rivers with lower DO.

(C) The survival rate of eels drops rapidly if the pH of the environmental water falls below 4 (Jellyman & Harding, 2014). Thus, we expected eel abundance to be lower in rivers with lower pH.

(D) Eels forage primarily on macrobenthic animals (Denis et al., 2022) and micro-invertebrates (Bardonnet & Riera, 2005), and they select habitats with more prey (Itakura et al., 2015). Thus, we expected eels to be more abundant in rivers with more prey animals. In addition, the abundance of micro-invertebrates correlates with electrical conductivity (EC) and turbidity (Kefford,1998; Henley et al., 2000), so we incorporated paths from EC and turbidity to eel CPUE.

(E) Eels prefer microhabitats with coarse substrates (Christoffersen et al., 2018). Thus, we expected eels to be more abundant in rivers with a large substrate grain size.

(F) Eels prefer locations with relatively deeper water (Domingos et al., 2006) and a relatively slower water flow (Kumai et al., 2021). Thus, we expected eels to be more abundant in rivers with greater depth and slower flow.

(G) Given that the upstream migration of glass eels arriving at the coast, and potential habitat shifts both among rivers and between rivers and the coastal zone, may be influenced by the size of the target river and adjacent rivers, we incorporated paths from the watershed area of each into our model.

(H) Since higher water temperatures are known to increase the catchability of eels using electrofishing (Degerman et al., 2019), we expected that CPUE would be higher when the water temperature at the time of the capture survey was higher.

(I) We expected rivers located where a large number of glass eels arrived at the coast to have a higher abundance of eels.

(J) High water temperature decreases DO levels and pH (Singh et al., 2004) and increases concentrations of dissolved nutrients (Ferreira and Chauvet, 2011) and the abundance of freshwater macro/micro-organisms (Daufresne et al., 2004). Thus, we expected summer water temperature to affect DO levels, pH, EC, turbidity, and macrobenthic animals.

(K) An increase of turbidity suppresses photosynthesis (Dokulil, 1994), and an increase of dissolved organic matter leads to a decrease in pH (Buffam et al., 2007) and an increase in the abundance of freshwater organisms (Salen-Picard et al., 2002). Thus, we expected turbidity to affect DO levels, pH, and the density of macrobenthic animals.

(L) Low pH has negative effects on the physiology of organisms (Schindler, 1988). Thus, we expected pH levels to affect the density of macrobenthic animals.

(M) Extreme cold can disrupt the physiology of organisms (Volkoff & Rønnestad, 2020). Thus, we expected winter water temperature to affect the density of macrobenthic animals.

(N) As the discharge from rivers increases, DO levels (Zhong et al., 2021) and turbidity (Goransson et al., 20013) increase. Thus, we expected water current velocity to affect DO levels and turbidity.

(O) Hypoxia causes mortality and modifies animal habitat selection (Breitburg, 2002). Thus, we expected low DO levels to affect the abundance of microbenthic animals.

(P) Eutrophication leads to an increase in the biomass of phytoplankton and consumer species, oxygen depletion, and a decrease in water transparency (Smith & Schindler, 2009). Thus, we expected EC to affect the density of microbenthic animals, DO levels, pH, and turbidity.

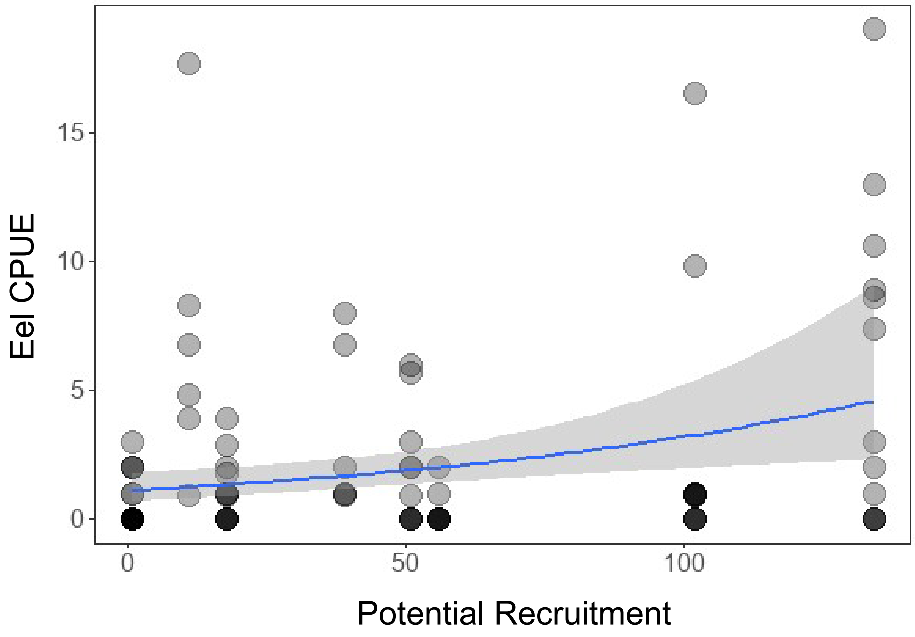

Fig. S2. Relationship between potential recruitment and eel CPUE. A generalized linear model assuming a negative binomial error distribution was fitted with CPUE as the response variable and potential recruitment as the explanatory variable ($R^2 = 0.09$, $P = 0.003$; Grey shading: 95% confidence interval). The residuals from this regression, representing CPUE adjusted for potential recruitment, were used in Figure 3b and Figure S4.

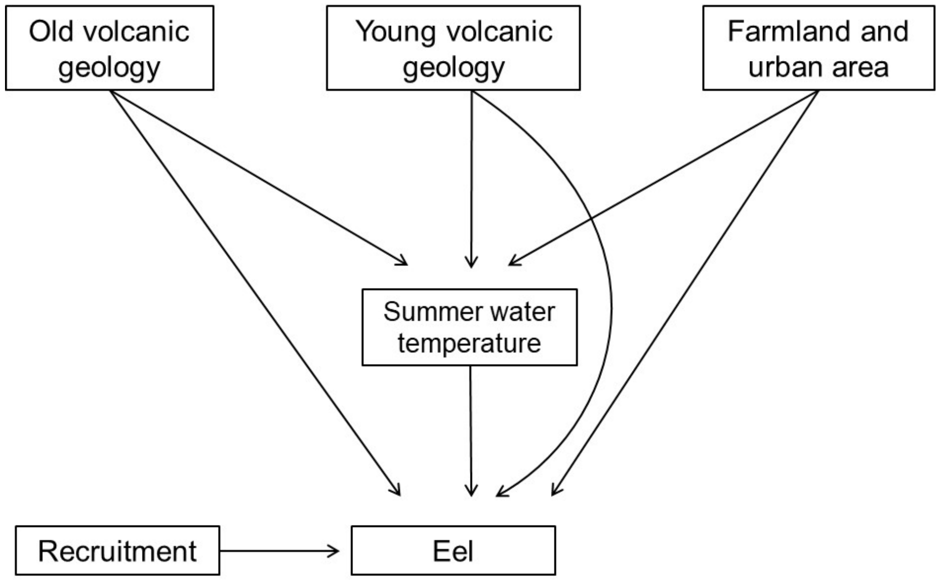

Fig. S3. Hypothesized causal network relationships between eel CPUE, three geographic variables, summer water temperature, and the degree of potential recruitment.

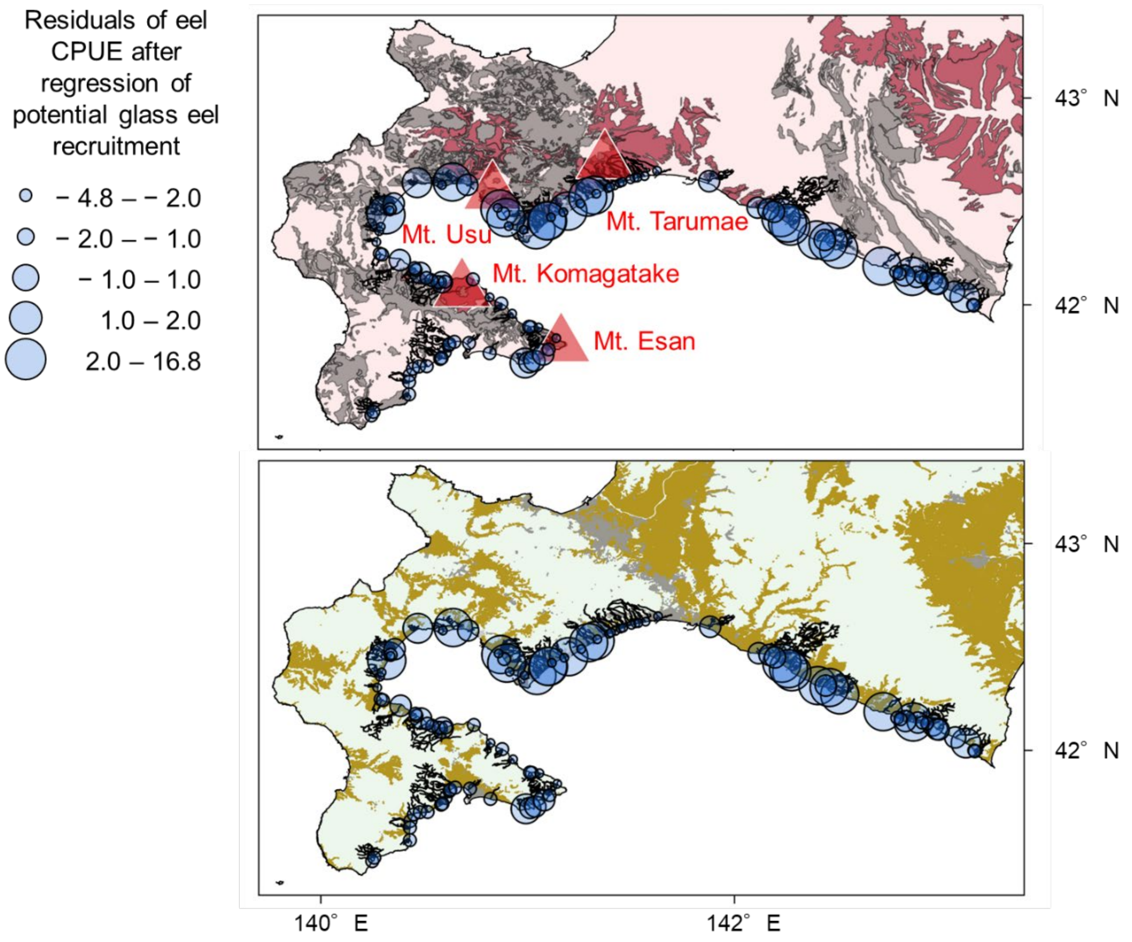

Fig. S4. Geographic features and abundance of Japanese eels. Circle size indicates the residuals of eel CPUE obtained by the regression of glass eel recruitment (see Fig. S2). Spatial distribution patterns of active volcanos and volcanic geology in southern Hokkaido (Upper panel). Areas shaded red are characterized by young volcanic geology, areas shaded gray are characterized by old volcanic geology, and active volcanoes are indicated by red triangles. Farmland and urban areas in southern Hokkaido. Farmland is indicated by brown shading, and urban areas are colored gray (Lower panel).

Table S1. Summary of the abiotic and biotic characteristics of the studied rivers.

Variables	Mean	Median	SD	Max	Min
Number of eels	2.1	0.0	3.9	19.0	0.0
Capture survey time (minutes)	62.3	61.0	2.8	78.0	60.0
Eel CPUE (number/hour)	2.1	0.0	3.8	19.0	0.0
Potential recruitment	46.5	38	47.8	133.0	0.0
**Environmental conditions**					
Summer water temperature (°C)	19.9	19.8	2.7	25.6	13.6
Winter water temperature (°C)	1.5	0.8	1.7	7.2	0.0
Water temperature at capture survey (°C)	18.9	18.5	3.2	27.0	12.0
pH	6.8	6.8	0.6	9.7	4.9
DO (mg/L)	8.2	8.2	0.6	9.7	5.9
EC (mS/m)	15.3	12.9	11.3	110.0	6.5
Turbidity (NTU)	3.9	2.9	3.3	15.3	0.1
Water depth (cm)	33.8	32.9	13.0	65.3	10.1
Substrate size	2.6	2.6	0.8	5.5	1.03
Water current velocity (m/s)	19.4	17.0	10.9	46.5	4.8
Density of macrobenthic animals	55.7	38.0	54.3	364.0	0.0
Size of target river (km^2)	26.2	15.2	33.6	287.4	1.5
Size of neighboring river (km^2)	41.3	12.5	68.6	340.5	1.2
**Geographic features**					
Sedimentary geology (%)	51.4	46.7	35.8	100.0	0.0
Old volcanic geology (%)	30.9	21.9	32.4	100.0	0.0
Young volcanic geology (%)	16.4	0	27.5	98.3	0.0
Farmland and urban areas (%)	13.9	9.5	14.5	77.1	0.0
Forest (%)	82.9	86.5	15.9	100.0	18.8
Air temperature (°C)	20.7	20.7	0.7	22.5	18.4
Precipitation (mm)	181.1	178.7	21.2	238.7	138.2
Solar radiation (MJ/m^2)	14.7	14.8	0.6	16.0	13.4

Table S2. Summary of the SEM incorporating eel CPUE, 13 environmental variables, and the degree of glass eel recruitment. Correlated factors are indicated (variables preceded by ~~) in the middle part of the table. Coefficients of determination (R^2) of models explaining the response variables are listed at the bottom of the table. Significant effects ($P < 0.05$) are indicated by bold text. The model is presented in Fig. 3

Response variable	Predictor variable	Estimate	Standard Error	P-value	β
**Eel CPUE**	**Summer water temperature**	**0.26**	**0.10**	**0.013**	**0.27**
Eel CPUE	Winter water temperature	-0.10	0.18	0.57	-0.051
Eel CPUE	Water temperature at capture survey	0.081	0.075	0.28	0.10
Eel CPUE	pH	0.28	0.47	0.55	0.063
Eel CPUE	DO	-0.34	0.31	0.27	-0.080
Eel CPUE	EC	-0.70	0.48	0.14	-0.12
Eel CPUE	Turbidity	0.24	0.19	0.21	0.11
Eel CPUE	Water depth	0.016	0.016	0.33	0.082
Eel CPUE	Substrate size	0.070	0.20	0.73	0.022
Eel CPUE	Water current velocity	0.012	0.021	0.56	0.051
Eel CPUE	Density of macrobenthic animals	-0.0007	0.0033	0.84	-0.014
Eel CPUE	Size of target river	0.045	0.22	0.83	0.016
Eel CPUE	Size of neighboring river	0.016	0.13	0.90	0.0083
**Eel CPUE**	**Potential recruitment**	**0.018**	**0.0039**	**<0.001**	**0.33**
**pH**	**Summer water temperature**	**0.054**	**0.017**	**0.0015**	**0.26**
**pH**	**EC**	**0.68**	**0.10**	**<0.001**	**0.54**
pH	Turbidity	0.025	0.04	0.54	0.048
DO	Summer water temperature	-0.034	0.025	0.17	-0.15
DO	EC	0.19	0.14	0.18	0.14
**DO**	**Turbidity**	**-0.15**	**0.058**	**0.0090**	**-0.28**
DO	Water current velocity	0.0015	0.0061	0.81	0.027
**EC**	**Summer water temperature**	**0.049**	**0.016**	**0.0020**	**0.30**
Turbidity	Summer water temperature	-0.0055	0.042	0.90	-0.014
**Turbidity**	**EC**	**0.56**	**0.24**	**0.02**	**0.23**
**Turbidity**	**Water current velocity**	**0.035**	**0.010**	**<0.001**	**0.35**
**Density of macrobenthic animals**	**Summer water temperature**	**0.16**	**0.040**	**<0.001**	**0.21**
Density of macrobenthic animals	Winter water temperature	0.046	0.076	0.54	0.029
Density of macrobenthic animals	pH	-0.14	0.21	0.51	-0.038
Density of macrobenthic animals	DO	0.099	0.16	0.53	0.029
Density of macrobenthic animals	EC	0.17	0.24	0.47	0.037
**Density of macrobenthic animals**	**Turbidity**	**-0.18**	**0.080**	**0.021**	**-0.098**
**~~Summer water temperature**	**~~Winter water temperature**	**-0.46**		**<0.001**	**-0.46**
**~~Summer water temperature**	**~~Water temperature at**	**0.69**		**<0.001**	**0.69**

	**capture survey**			
**~~Winter water temperature**	**~~EC**	**0.22**	**0.013**	**0.22**
**~~Winter water temperature**	**~~TURB**	**0.25**	**0.0057**	**0.25**
**~~Winter water temperature**	**~~DO**	**-0.21**	**0.017**	**-0.21**
**~~pH**	**~~DO**	**0.42**	**<0.001**	**0.42**
**~~pH**	**~~Water current velocity**	**0.18**	**0.034**	**0.18**
**~~pH**	**~~Water depth**	**-0.19**	**0.026**	**-0.19**
**~~pH**	**~~Size of neighboring river**	**-0.23**	**0.011**	**-0.23**
**~~TURB**	**~~Potential Recruitment**	**0.23**	**0.011**	**0.23**
**~~TURB**	**~~Size of neighboring river**	**0.30**	**0.0011**	**0.30**
**~~Water depth**	**~~Water current velocity**	**-0.44**	**<0.001**	**-0.44**
**~~Density of macrobenthic animals**	**~~Size of target river**	**0.29**	**0.0013**	**0.29**
**R^2**				
**Eel CPUE**	**0.51**			
**pH**	**0.45**			
**DO**	**0.08**			
**EC**	**0.09**			
**Turbidity**	**0.18**			
**Density of macrobenthic animals**	**0.27**			

Table S3. Summary of the results of the multiple regression analysis examining the relationship between summer water temperature and five geographic variables. Significant effects ($P < 0.05$) are indicated by bold text.

	Estimate	Standard Error	*t*-value	*P*-value	β
Intercept	22.583	5.832	3.872	< 0.001	
**Old volcanic geology**	**–0.024**	**0.006**	**–3.804**	**< 0.001**	**–0.293**
**Young volcanic geology**	**–0.065**	**0.008**	**–7.983**	**< 0.001**	**–0.663**
**Farmland and urban areas**	**0.042**	**0.014**	**3.054**	**0.003**	**0.236**
Precipitation	–0.006	0.011	–0.582	0.562	–0.050
Solar radiation	–0.020	0.346	–0.057	0.954	–0.004

Table S4. Summary of the results of the SEM incorporating eel CPUE, summer water temperature, degree of glass eel recruitment, and three geographic variables. The coefficients of determination (R^2) of models explaining the response variables are provided at the bottom of the table. Significant effects ($P < 0.05$) are indicated by bold text. Model is presented in Fig. 5

Response variable	Predictor variable	Estimate	Standard Error	P-value	β
**Eel CPUE**	**Summer water temperature**	**0.30**	**0.086**	**< 0.001**	**0.40**
**Eel CPUE**	**Potential recruitment**	**0.019**	**0.0042**	**< 0.001**	**0.45**
Eel CPUE	Old volcanic geology	-0.0064	0.0061	0.30	-0.11
Eel CPUE	Young volcanic geology	-0.0070	0.0094	0.46	-0.097
Eel CPUE	Farmland and urban areas	-0.0038	0.012	0.74	-0.028
**Summer water temperature**	**Old volcanic geology**	**-0.024**	**0.0062**	**< 0.001**	**-0.29**
**Summer water temperature**	**Young volcanic geology**	**-0.067**	**0.0071**	**< 0.001**	**-0.69**
**Summer water temperature**	**Farmland and urban areas**	**0.043**	**0.013**	**0.0015**	**0.23**
R^2					
Eel CPUE	0.43				
Summer water temperature	0.51				

Additional References

Fukuda, N., Kuroki, M., Shinoda, A., Yamada, Y., Okamura, A., Aoyama, J., & Tsukamoto, K. (2009). Influence of water temperature and feeding regime on otolith growth in *Anguilla japonica* glass eels and elvers: does otolith growth cease at low temperatures? *Journal of Fish Biology*, 74, 1915–1933. https://doi.org/10.1111/j.1095-8649.2009.02287.x

Han, Y.-S., Yambot A. V., Zhang, H., & Hung, C. L. (2012). Sympatric spawning but allopatric distribution of Anguilla japonica and Anguilla marmorata: Temperature-and oceanic current-dependent sieving. *PLOS ONE*, 7, e37484. https://doi.org/10.1371/journal.pone.0037484

Edeline, E., Lambert, P., Rigaud, C., & Elie. P. (2006). Effects of body condition and water temperature on *Anguilla anguilla* glass eel migratory behavior. *Journal of Experimental Marine Biology and Ecology*, 331, 217–225. https://doi.org/10.1016/j.jembe.2005.10.011

Rowchai, S., Chiba, K., & Hirano, R. (1986). Influence of dissolved oxygen on growth of young eel. *Bulletin of the Japanese Society of Scientific Fisheries*, 52, 597–607. https://doi.org/10.2331/suisan.52.597

Jellyman, P. G., & Harding, J. S. (2014). Variable survival across low pH gradients in freshwater fish species. *Journal of Fish Biology*, 85, 1746–1752. https://doi.org/10.1111/jfb.12497

Denis, J., Rabhi, K., Le Loc'h, F., Ben Rais Lasram, F., Boutin, K., Kazour, M., … Amara, R. (2022). Role of Estuarine Habitats for the Feeding Ecology of the European Eel (*Anguilla anguilla* L.). *PLoS ONE*, 17, e0270348. https://doi.org/10.1371/journal.pone.0270348

Bardonnet, A., & Riera, P. (2005). Feeding of glass eels (*Anguilla anguilla*) in the course of their estuarine migration: new insights from stable isotope analysis. *Estuarine Coastal and Shelf Science*, 63, 201–209. https://doi.org/10.1016/j.ecss.2004.11.009

Itakura, H., Kaino, T., Miyake, Y., Kitagawa, T., & Kimura, S. (2015). Feeding, condition, and abundance of Japanese eels from natural and revetment habitats in the Tone River, Japan. *Environmental Biology of Fishes*, 98, 1871–1888. https://doi.org/10.1007/s10641-015-0404-6

Kefford B. J. (1998) The relationship between electrical conductivity and selected macroinvertebrate communities in four river systems of south-west Victoria, Australia. *International Journal of Salt Lake Research*, 7, 153–170. https://doi.org/10.1007/BF02441884

Henley, W. F., Patterson, M. A., Neves. R. J., & Lemlym, A. D. (2000). Effects of

sedimentation and turbidity on lotic food webs: a concise review of natural resource managers. *Reviews in Fisheries Science*, 8, 125–139. https://doi.org/10.1080/10641260091129198

Christoffersen, M., Svendsen, J. C., Kuhn, J. A., Nielsen, A., Martjanova, A., & Støttrup, J. G. (2018). Benthic habitat selection in juvenile European eel *Anguilla anguilla*: Implications for coastal habitat management and restoration. *Journal of Fish Biology*, 935, 996–999. https://doi.org/10.1111/jfb.13807

Domingos, I., Costa, J. L., & Costa, M. J. (2006). Factors determining length distribution and abundance of the European eel, *Anguilla anguilla*, in the River Mondego (Portugal). *Freshwater Biology*, 51, 2265–2281. https://doi.org/10.1111/j.1365-2427.2006.01656.x

Kumai, Y., Kuroki, M., & Morita, K. (2021). Influence of environmental parameters on habitat use by sympatric freshwater eels Anguilla marmorata and Anguilla japonica on Yakushima Island, Japan. Canadian Journal of Zoology, 99, 1020–1027. https://doi.org/10.1139/cjz-2021-0125

Singh, K. P., Malik, A., Mohan, D., & Sinha, S. (2004). Multivariate statistical techniques for the evaluation of spatial and temporal variations in water quality of Gomti River (India) - A case study. *Water Research*, 38, 3980–3992. https://doi.org/10.1016/j.watres.2004.06.011

Ferreira, V., & Chauvet, E. (2011). Synergistic effects of water temperature and dissolved nutrients on litter decomposition and associated fungi. *Global Change Biology*, 17, 551–564. https://doi.org/10.1111/j.1365-2486.2010.02185.x

Daufresne, M., Roger, M. C., Capra, H. & Lamouroux, N. (2004). Long-term changes within the invertebrate and fish communities of the Upper Rhone River: effects of climatic factors. *Global Change Biology*, 10, 124–140. https://doi.org/10.1046/j.1529-8817.2003.00720.x

Dokulil, M. T. (1994). Environmental control of phytoplankton productivity in turbulent turbid systems. *Hydrobiologia*, 289, 65–72. https://doi.org/10.1007/BF00007409

Buffam, I., Laudon, H., Temnerud, J., Mörth, C.-M., & Bishop, K. (2007). Landscape-scale variability of acidity and dissolved organic carbon during spring flood in a boreal stream network. Journal of Geophysical Research: *Biogeosciences*, 112, G01022. https://doi.org/10.1029/2006JG000218

Salen-Picard, C., Darnaude, A. I., Arlhac, D., & Harmelin-Vivien, M. I. (2002). Fluctuations of macrobenthic populations: a link between climate-driven river run-off and sole fishery yields in the Gulf of Lions. *Oecologia*, 133, 380–388. https://doi.org/10.1007/s00442-002-1032-3

Schindler, D. S. (1988). Effects of acid rain on freshwater ecosystems. *Science*, 239,149–

157. https://www.jstor.org/stable/1700443

Volkoff, H., & Rønnestad, I. (2020). Effects of temperature on feeding and digestive processes in fish. Temperature, 7, 307–320. https://doi.org/10.1080/23328940.2020.1765950

Zhong, M., Liu, S., Li, K., Jiang, H., Jiang, T., & Tang, G. (2021). Modeling Spatial Patterns of Dissolved Oxygen and the Impact Mechanisms in a Cascade River. *Frontiers in Environmental Science*, 9, 781646. https://doi.org/10.3389/fenvs.2021.781646

Goransson, G., Larson, M., & Bendz, D. (2013). Variation in turbidity with precipitation and flow in a regulated river system—River GötaÄlv, SW Sweden. *Hydrology and Earth System Sciences*, 17, 2529–2542. https://doi.org/10.5194/hess-17-2529-2013

Breitburg, D. L. (2002). Effects of hypoxia, and the balance between hypoxia and enrichment, on coastal fishes and fisheries. *Estuaries,* 25,767–781. https://doi.org/10.1007/BF02804904

Smith, V. H., and Schindler, D. W. 2009. Eutrophication science: where do we go from here?" *Trends in Ecology & Evolution*, 24, 201–207. https://doi.org/10.1016/j.tree.2008.11.009
